# GENETICS 2025 Reviewer Index

**DOI:** 10.1093/genetics/iyaf231

**Published:** 2025-12-10

Below is a list of individuals who have reviewed manuscripts for GENETICS during the preparation of Volumes 229, 230, and 231 (2025). The aim of GENETICS is to communicate significant research. To succeed, we must recognize what is significant, making sure the presentation is accurate, unambiguous, and easily readable. For this, we depend heavily upon our reviewers, who perform this indispensable service without reward. The quality of the journal strongly reflects the quality of their efforts. We apologize to any reviewer whose name has been inadvertently omitted, and we are sincerely grateful to all.

Abalde-Atristain, Leire

Abbott, Allison L.

Abbott, Jessica K.

Ables, Elizabeth T.

Abood, Abdullah

Abrash, Elizabeth

Abu Awad, Diala

Achaz, Guillaume

Adelman, Karen

Adelman, Zach N.

Adilieje, Chioma M.

Afsharyan, Nazanin

Agarwal, Priti

Agha, Husain

Agrawal, Aneil F.

Ahlawat, Neetika

Ahlinder, Jon

Ahmad, Kami

Ahmed, Osama M.

Ahmed, Shawn

Ahmed-Braimah, Yasir H.

Ahringer, Julie

Aiello, Allison

Akakpo, Roland

Akera, Takashi

Alani, Eric

Albà, Mar

Albertson, Craig

Aldulijan, Ibrahim

Alexander, Helen K.

Alexandre, Arthur

Ali, Ahmad

Alkhawaja, Abdalla

Allen, Jessica A.

Alonso-Blanco, Carlos

Alphey, Luke

Altman, Arie

Amir, Ariel

Amodeo, Amanda

Amodeo, Amanda A.

An, Shiheng

Andersen, Erik C.

Anderson, Alexander R.

Anderson, Faith

Anderson, Matthew Z.

Andersson, Dan I.

Andorf, Carson M.

Andrews, Brenda J.

Anisimova, Maria

Antal, Tibor

Apfeld, Javier

Aquilante, Christina

Aquino Nunez, Wendy

Aravin, Alexei

Arbeitman, Michelle N.

Arbelaez, Juan D.

Archambeault, Sophie

Arella, Davide

Arends, Danny

Arge, Luis W.

Arjas, Arttu

Armstrong, Alissa

Arndt, Karen M.

Arnold, Brian

Arnosti, David N.

Arribere, Joshua A.

Arur, Swathi

Asahina, Kenta

Aschard, Hugues

Ashbrook, David G.

Askjaer, Peter

Aubé, Simon

Ausin, Israel

Avelino, Camila C.

Ayala, Diego

Aydin, Selcan

Aylor, David L.

Ayroles, Julien F.

Babst, Markus

Bach, Erika A.

Bachand, Francois

Bachtrog, Doris

Backström, Niclas

Badet, Thomas

Badonyi, Mihaly

Baer, Charles F.

Bageritz, Josephine

Bähler, Jürg

Bahn, Yong-Sun

Bailey, Nathan W.

Baird, Stuart J.

Bancic, Jon

Bandyadka, Shruthi

Bank, Claudia

Bannasch, Danika

Baños, Hector

Bao, Zhirong

Barber, Annika

Barger, Sarah R.

Barlow, Miriam

Barott, Katie L.

Barr, Maureen M.

Barrales, Ramon R.

Barratt, Kristen S.

Barrett, Rowan D.

Barton, John P.

Barton, Nicholas H.

Basak, Piyali

Baserga, Susan

Bass, Chris

Bastian, Frederic Bastian

Basu, Saonli

Bataillon, Thomas

Bateman, Alex

Bateman, Jack R.

Batista, Lorena

Baud, Amelie

Baugh, Ryan

Bavik, Linnea

Baym, Michael

Bazzini, Ariel A.

Beaumont, Mark

Becker, Peter B.

Becker, Thomas

Beckouet, Frederic

Beebe, Nigel W.

Beets, Isabel

Behrman, Emily

Bell, Stephen

Bellaiche, Johanns

Bellen, Hugo J.

Beltrao, Pedro

Bénard, Claire Y.

Bendesky, Andres

Bendixsen, Devin P.

Bennett, Richard J.

Bensasson, Douda

Benyamin, Beben

Berger, James

Bergeron, Lucie A.

Bergland, Alan

Bergland, Alan O.

Bergman, Casey M.

Berube, Benjamin

Berv, Jacob

Bessereau, Jean-Louis

Bessho, Kazuhiro

Betancourt, Andrea

Betran, Esther

Bett, Kirstin E.

Bhalla, Needhi

Bhamidipati, Sridevi

Bhandari, Prashant

Bhar, Monmita

Bhat, Umer S.

Bhattacharjee, Shatabdi

Bhattacharya, Aniket

Bhattacharya, Arjun

Bhattarai, Upendra R.

Bhering, Leonardo L.

Bickel, Sharon E.

Bielas, Stephanie

Bielinsky, Anja K.

Biggins, Sue

Bijma, Piter

Billiard, Sylvain

Billmyre, Katie

Birchler, James A.

Bird, Amanda

Blagojević, Jelena

Blake, Victoria C.

Bleichert, Franziska

Bloom, Jesse D.

Bloom, Joshua S.

Bloom, Kerry S.

Blumenstiel, Justin P.

Blundell, Jamie

Bobay, Louis-Marie

Boddy, Nick

Boeke, Jef D.

Boer, Martin P.

Bolla, Jean M.

Bolognesi, Benedetta

Bolterstein, Elyse

Boman, Jesper

Bomblies, Kirsten

Bondy-Denomy, Joseph

Boone, Charles

Borges, Rui

Börner, G. Valentin

Borreguero-Munoz, Nerea

Bose, Banabithi

Bouchard-cote, Alexandre

Boulding, Elizabeth G.

Bourke, Peter M.

Boyle, Gabriel E.

Bozic, Ivana

Bracewell, Ryan R.

Bradburd, Gideon S.

Brandvain, Yaniv

Branzei, Dana

Brar, Gloria A.

Braun, Robert E.

Brem, Rachel B.

Brenner, Charles

Breuker, Casper

Breuza, Lionel

Brewer, Bonita J.

Briggs, Scott D.

Brill, Julie A.

Britt, Anne

Brockhurst, Michael

Broday, Limor

Broman, Karl W.

Brow, David A.

Brown, Andre E.

Brown, Grant W.

Browning, Brian L.

Bruford, Elspeth

Brughmans, Tom

Bryson Richardson, Robert

Buckler, Edward S.

Buckling, Angus

Buechner, Matthew

Buell, C. Robin

Buffalo, Vince

Bukhari, Hassan

Bülow, Hannes E.

Bunter, Kim

Buratowski, Stephen

Bureau, Alexandre

Burini Kojin, Bianca

Burke, Molly

Burnetti, Anthony

Burns, Michael

Burow, Mark D.

Burtis, Kenneth C.

Buszczak, Michael

Butcher, Rebecca

Butler, Geraldine

Butler, Scott

Buttitta, Laura A.

Caballero, Armando

Cabernard, Clemens

Cabianca, Daphne

Cabugos, Leyla

Cagan, Alex

Cai, Haini N.

Cai, Haoran

Cai, Mingxuan

Cairns, Brad

Calus, Mario P.

Calvi, Brian R.

Cam, Hugh P.

Canario, Laurianne

Canman, Julie

Cannon, Ethalinda K.

Cao, Hongyuan

Cao, Wei

Carbone, Ignazio

Carme, Pascal

Carmi, Shai

Carr, Antony

Carr, Erin C.

Carrera, Ines

Carvunis, Anne-Ruxandra

Casale, Assunta Maria

Caselle, Michele

Casoni, Filippo

Castellano, David

Castellano, Luciana

Castric, Vincent

Castro, Victoria L.

Cavallaro, Amanda

Cavieres-Lepe, Javier

Cecere, Germano

Celen, Irem

Cepeda-Plaza, Marjorie

Chalmel, Frederic

Chambers, Moria

Champer, Jackson

Chanfreau, Guillaume

Chang, Christie S.

Chang, Michael

Chapman, Tracey

Chappidi, Sruthi

Charbon, Godefroid

Charlesworth, Brian

Chatterjee, Sumantra

Chauhan, Neeraj

Chavan, Ankita

Chavez, Daniela R.

Chayut, Noam

Chee, Peng W.

Chen, Changchun

Chen, Dawn S.

Chen, Fei

Chen, Jiawen

Chen, Jingxian

Chen, Ju

Chen, Nannan

Chen, Patrick

Chen, Peiwei

Chen, Wenbiao

Chen, Z. J.

Chen , Pao-Yang

Cheng, Xiaoheng

Cherbas, Peter T.

Cherra, Salvatore

Chesler, Elissa J.

Chesnokov, Igor

Cheung, Ching Lung

Cheverud, James M.

Chevin, Luis-Miguel

Chigweshe, Lorencia

Chikina, Maria

Chin-Sang, Ian

Chiolo, Irene

Chipman, Ariel

Chisholm, Andrew D.

Chitre, Apurva S.

Choe, Keith P.

Choudhary, Mukesh

Chougule, Kapeel

Chow, Clement Y.

Christensen, Ole F.

Christensen, Tim W.

Christie, Karen

Chung, George

Church, Michael

Churchman, Stirling

Ciosk, Rafal

Clare, Shaun J.

Claridge-Chang, Adam

Clark, Andrew G.

Claycomb, Julie M.

Clo, Josselin

Cochella, Luisa

Cohen, Ehud

Cohen, Paula E.

Collins, Kathleen

Comeron, Josep M.

Comfort, Nathaniel C.

Conibear, Elizabeth

Connallon, Tim

Conneely, Karen

Conneely, Karen N.

Conradt, Barbara

Consalez, Gian Giacomo

Contreras, Adrian

Cook, Jean

Cook, Kevin R.

Cook, Stuart

Coop, Graham

Cooper, Brandon S.

Cooper, Kimberly

Cooper, Mark

Cooper, Vaughn S.

Cope, Alexander

Copeland, Megan

Copenhaver, Gregory P.

Corbett-Detig, Russell B.

Cordell, Heather J.

Corringer, Pierre-Jean

Corrochano, Luis M.

Cortazar, Maria

Cortes, Katherina

Costa, Rodolfo

Costain, Gregory

Costanzo, Michael

Côté, Jacques

Cotto, Olivier

Courcelle, Justin

Courret, Cécile

Courtier-Orgogozo, Virginie

Courtot, Melanie

Cousins, Trevor

Coutinho-Budd, Jaeda

Cram, Erin J.

Crawford, Lorin

Cristescu, Melania E.

Cristofari, Gael

Crocker, Justin

Croll, Daniel

Cromie, Gareth A.

Crona, Kristina

Crosby, Andrew

Croset, Vincent

Crossa, José

Crown, K. N.

Cubillos, Francisco

Cui, Tianyu

Culotti, Joe G.

Cuomo, Christina A.

Curran, Sean P.

Cusanelli, Emilio

Cutler, David J.

Cutter, Asher D.

Cvijovic, Ivana

Czuppon, Peter

D'Angiolo, Melania

D'arcangelo, Gabriella

Daetwyler, Hans D.

Dai, Qi

Dai, Siyuan

Dai, Xihuimin

Damschroder, Deena

Danko, Charles

Danko, Charles G.

Das, Suman

De Bono, Mario

De Camilli, Pietro

De Cuevas, Margaret

De la Cova, Claire C.

De la Cruz, Jesús

De la Cruz Arguello, Ivan M.

De Leon, Natalia

De los Campos, Gustavo

De Maio, Nicola

De Manuel Montero, Marc

De Meaux, Juliette

De Muyt, Arnaud

De Ridder, Dick

De Villemereuil, Pierre

De Visser, Arjan

Dean, Antony M.

Dean, Matthew D.

Débarre, Florence

Debener, Thomas

Decroly, Thomas

DeGiorgio, Michael

Dekkers, Jack C.

DelaFuente, Javier

Delneri, Daniela

DeLuca, Steven

DeLuna, Alexander

Deng, Hong-Wen

Deng, Wu-Min

Deng, Yun

Deraje, Puneeth

Derbyshire, Mark C.

Des Marais, David

Desai, Michael M.

Desplan, Claude

Després, Philippe C.

Desselhaus, Thomas

Deutsch, Eric

Devi, Archana

DeWitt, William

Dey, Gautam

Dhawanjewar, Abhilesh

Di, Yazheng

Di Pietro, Antonio

Diaz Vazquez, Rebeca

Diaz-Garcia, Luis

Diaz-Papkovich, Alex

Dickinson, Daniel J.

Diepenbrock, Christine H.

Dijk, Aalt-Jan V.

DiNardo, Stephen

Diss, Guillaume

Divekar, Nikita

Doe, Chris Q.

Doeschl-Wilson, Andrea B.

Don Roo, Hyung

Donczew, Rafal

Dong, Emily

Dorsch, Michael

Dorus, Steve

Dowen, Jill M.

Draghi, Jeremy

Dragon, François

Drown, Melissa

Du, Jiawen

Du, Li-Lin

Duchaine, Thomas

Dudbridge, Frank

Dudley, Aimee M.

Dudycha, Jeff

Duester, Gregg

Dumont, Beth L.

Dunham, Alistair

Dunham, Maitreya J.

Durbin, Richard

Duret, Laurent

Duronio, Robert J.

Durvasula, Arun

Dus, Monica

Dutheil, Julien Y.

Dutta, Abishek

Duveau, Fabien

Dworkin, Ian

Dyer, Paul

Dyer, Sarah

Eckert, Andrew J.

Eckmann, Christian

Edery, Isaac

Edgar, Bruce A.

Edge, Michael D.

Edwards, David

Ehrenreich, Ian M.

Eide, David J.

Elaswad, Mohamed T.

Elena, Santiago F.

Elf, Johan

Ellis, David A.

Ellison, Shelby L.

Elmer, Kathryn

Elsadany, Muhammad

Elsik, Christine

Emanuele, Mike

Emerson, J. J.

Emery, Patrick

Endelman, Jeffrey B.

Enduru, Nitesh

Engebrecht, JoAnne

Engel, Stacia R.

Engelstädter, Jan

English, Stephanie

Ercan, Sevinç

Erickson, Priscilla A.

Eskandari, Milad

Eskin, Eleazar

Esvelt, Kevin

Etheridge, Alison M.

Evans, Kathryn S.

Everitt, Richard

Everman, Elizabeth R.

Eves-van den Akker, Sebastian

Ewe, Chee Kiang

Fallahshahroudi, Amir

Farmer, Andrew D.

Farrell, Colin

Faulkner, Geoffrey

Faure, Andre J.

Favero, Martina

Fay, David S.

Fay, Justin C.

Feder, Alison F.

Fedorka, Ken

Feingold, Eleanor

Félix, Marie-Anne

Feller, Anna

Feng, Qian

Feng, Siyuan

Ferguson, Betsy

Ferkey, Denise M.

Fernandes, Samuel B.

Fernando, Rohan

Ferrandon, Dominique

Ferree, Patrick M.

Ferreira, Clara

Ferreiro, Alejandro M.

Ferris, Martin T.

Ficklin, Stephen

Ficner, Ralph

Fields, Peter D.

Finnigan, Gregory C.

Fischer, Gilles

Fischer, Sylvia

Fisher, Kaitlin J.

Fishman, Lila

Fitch, David H.

Flatt, Thomas

Flegontov, Pavel

Fogel, Arielle S.

Folker, Eric

Fontanesi, Flavia

Foo, Jasmine

Forest, Thomas

Forien, Raphael

Forsburg, Susan L.

Foster, Peter

Foucher, Fabrice

Fouqueau, Louise

Fowler, Douglas M.

Fowler-Finn, Kasey

Fox, Donald T.

Francis, Michael

Frank, Chan

Frank, Steven A.

Franz, Alexander W.

Freire, Raimundo

Freund, Fabian

Fritsche-Neto, Roberto

Frøkjær-Jensen, Christian

Fu, Daolin

Fullerton, Stephanie M.

Fung, Jennifer

Gabaldón, Toni

Gadal, Olivier

Galardini, Marco

Gallicchio, Lorenzo

Gamble, Tony

Ganguly, Anindya

Ganley, Austen R.

Gao, Hongding

Gao, Ziyue

Garnier, Jimmy

Garrison, Erik

Garsin, Danielle A.

Gartner, Anton

Garton, Fleur

Garud, Nandita

Gasch, Audrey P.

Gatfield, David

Gatti, Daniel M.

Gatti, Maurizio

Gaudet, Pascale

Gaudriault, Sophie

Gaunt, Tom

Gautel, Mathias

Gearhart, Micah D.

Geck, Renee C.

Gehan, Malia

Geli, Vincent

Gent, Jonathan I.

Georgiev, Pavel

Gerrish, Philip J.

Geyer, Pamela K.

Ghose, Piya

Ghosh, Neha

Gianola, Daniel

Gibb, Andrew

Gibbons, John G.

Gibson, Matt

Gifford, Danna R.

Gilbert, Kimberly J.

Gilchrist, Michael A.

Gill, Dipender

Gill, Matthew S.

Gillespie, Zoe E.

Gillman, Jason D.

Gilson, Eric

Giniger, Edward

Gitler, Aaron

Gladieux, Pierre

Glater, Elizabeth E.

Glauser, Dominique A.

Glémin, Sylvain

Gligorovski, Vojislav

Gnanadesikan, Gitanjali

Gnanapragasam, Niranjani

Gódia, Marta

Godwin, Ian

Goldberg, Amy

Goldman, Gustavo H.

Gomer, Richard

Gomez-Cano, Fabio A.

Gonzalez, Josefa

González-Diéguez, David O.

Good, Benjamin H.

Goode, Bruce L.

Goodman, Lindsey

Goodman, Miriam B.

Goodstein, David M.

Goodwillie, Carol

Gordo, Isabel

Gordon, Kacy L.

Gordon, Michael D.

Gore, Michael A.

Gorjanc, Gregor

Gorman, Maureen

Gottschalk, Alexander

Gou, Liangke

Goubert, Clement

Gould, Kathleen L.

Goutte, Caroline

Graf, Ralph

Graff, Misa

Graham, JInko

Grant, Nkrumah

Gravel, Simon

Green, Andrew

Green, Llewellyn

Green, Sabrina

Greene, Carol

Greene, Devin

Greenfield, Michael D.

Greenspoon, Philip

Greenwald, Iva

Greer, Eric L.

Greider, Carol W.

Greiss, Sebastian

Gresham, David

Grieshop, Karl

Griffith, Leslie C.

Griffiths, Joanna

Grishok, Alla

Griswold, Cortland K.

Groen, Simon C.

Grosshans, Helge

Grutenko, Nataly

Gu, Sam G.

Gu, Sam Guoping

Guang, Shouhong

Gueguen, Laurent

Guet, Calin C.

Guha Majumdar, Sayanti

Guilherme Pereira, Caio

Guillaume, Frédéric

Gumienny, Tina L.

Gunasekaran, Deepika

Günesdogan, Ufuk

Gunnarsson, Árni Freyr

Gunnarsson, Einar Bjarki

Guo, Bing

Guo, Xinyi

Guo, Yungui

Guschanski, Katerina

Gutenkunst, Ryan N.

Gutierrez, Lucía

Gymrek, Melissa

Haag, Christoph R.

Haag, Eric S.

Haase, Max

Haase, Max A.

Haasl, Ryan

Hadany, Lilach

Hadfield, Jarrod D.

Hadjur, Suzana

Haendel, Melissa

Hafen, Ernst

Hagen, Iain

Haghani, Amin

Hajnal, Alex

Halder, Georg

Halfon, Marc S.

Hall, James

Hall, Mark C.

Hall, Sarah E.

Hallatschek, Oskar

Hammell, Christopher M.

Hammond, Thomas M.

Hammond-Kosack, Kim E.

Hamza, Iqbal

Han, Jae Yong

Han, Min

Han, Myeonghoon

Han, Shunhua

Hanlon, Stacey L.

Hanly, Joseph J.

Hanna-Rose, Wendy

Hansen, Dave

Hanson, Mark A.

Hardin, Jeff

Harfouche, Antoine L.

Harigaya, Yuriko

Hariharan, Iswar K.

Hariri, Sara

Harms, Henrik

Harpack, Arbel

Harrero, Salva

Harrington, Lea A.

Harris, Kelley

Harris, Steven D.

Harrison, Ellie

Hart, Anne C.

Hartenstein, Volker

Hartmann-Petersen, Rasmus

Hashimshony, Tamar

Hastings, Philip J.

Havird, Justin C.

Hay, Bruce A.

Hayward, Laura K.

Hazra, Ujani

He, Bin Z.

He, Mingze

Hedrick, Philip W.

Heifetz, Yael

Hein, Jotun J.

Heinig, Matthias

Helfrich-Förster, Charlotte

Henderson, Ian R.

Hendrickson, Heather L.

Henikoff, Steven

Heppert, Jennifer K.

Herman, Christophe

Hermann, Greg J.

Herriage, Hunter C.

Hieter, Philip

Hiller, Michael

Hilliard, Massimo A.

Hilliker, Angie

Hime, Gary

Hinch, Anjali

Hinnebusch, Alan G.

Hirsh, Jay

Hisamoto, Naoki

Hittinger, Chris T.

Hobolth, Asger

Hochstrasser, Mark

Hoffman, Ellen

Hogan, Deborah A.

Hoge, Carla

Hollick, Jay B.

Hopper, Anita K.

Hormozdiari, Fereydoun

Horvath, Steve

HoSang, Daniel M.

Hosken, David J.

Hotchkiss, Jade

Hou, Jing

Houben, Andreas

Houle, David

Houseley, Jon

Howe, Douglas G.

Hsiao, Yi-Wen

Hu, Chang-Deng

Hu, Xianghong

Huang, Jun

Huang, Kerui

Huang, Xuehui

Huang, Yuheng

Hubbard, E. Jane A.

Huber, Christian D.

Hughes, David

Hughes, Stacie E.

Hulse-Kemp, Amanda

Hundley, Heather A.

Hunter, Craig P.

Hunter, Martha

Hurst, Laurence D.

Hussain, Waseem

Huttenhower, Curtis

Idnurm, Alexander

Igic, Boris

Igler, Claudia

Ignatieva, Anastasia

Iino, Yuichi

Ikele, Chinyere M.

Ikura, Mitsuhiko

Immler, Simone

Ingalls, Brian

Inoue-Murayama, Miho

Inwood, Sarah

Iqbal, Sumaiya

Ishihara, Takeshi

Isidro Sánchez, Julio

Ismat, Afshan

Iyengar, Atulya

Iyer, Shruti V.

Izadi, Mahmoud

Izquierdo, Paulo

Jackson, F. R.

Jafar-Nejad, Hamed

Jagadish, Krishna

Jaiswal, Manish

Jakšić, Ana M.

Jalal, Deena

James-Zorn, Christina H.

Jankowsky, Eckhard

Jannink, Jean-Luc

Jaramillo-Lambert, Aimee

Jarquin, Diego

Jay, Paul

Jaynes, James B.

Jenner, Richard

Jensen, Jeffrey D.

Jeon, Jong Yoon

Jermiin, Lars S.

Jia, Songtao

Jiang, Min-Zhi

Jiang, Rui

Jiang, Yong

Jiggins, Chris D.

Jin, Jin

Jin, Yui

Johannesson, Hanna

Johansen, Katja

Johnson, Alexander D.

Johnson, Arlen W.

Johnson, Quinn

Johnston, Laura A.

Johnston, Susan E.

Johri, Parul

Jones, Andrew

Jones, Felicity

Jorde, Lynn B.

Jose, Antony M.

Joseph, Julien

Joyce, Eric

Juenger, Thomas E.

Jun, Suckjoon

Jung, Sook

Junier, Ivan

Kadener, Sebastian

Kaduskar, Bhagyashree D.

Kai, Toshie

Kajungiro, Redempta A.

Kane, Daniel P.

Kaneko, Kunihiko

Kang, Guolian

Kang, Yunsik

Karageorgiou, Charikleia

Karhunen, Ville

Karlsson, Elinor

Karp, Xantha

Kasho, Kazutoshi

Kasimatis, Katja R.

Kassis, Judith A.

Kastaniotis, Alexander J.

Katariya, Rajnandani

Katayama, Tsutomu

Katju, Vaishali

Katsura, Isao

Katz, David J.

Kaun, Karla R.

Kaur, Maninder

Kaur, Rupinder

Kayondo, Siraj I.

Keating, Amy

Keiper, Brett D.

Keith, Alasdair

Kelleher, Jerome

Keller, Matthew C.

Kelley, Joanna L.

Kelly, John K.

Kern, Andrew D.

Kerr, Benjamin

Ketting, René

Khadilkar, Rohan J.

Khammash, Mustafa

Khan, Rehan

Khetchoumian, Konstantin

Kidd, Jeff

Kiktev, Denis A.

Kim, Byung-Eun

Kim, Byunghyuk

Kim, Dennis H.

Kim, Jaehee

Kim, Tae Hyun

King, Aaron

King, Elizabeth G.

Kirkpatrick, Mark

Kitaoka, Maiko

Kivikoski, Mikko

Klaembt, Christian

Klasson, Lisa

Klein, Jason C.

Klein, Patricia E.

Klingenberg, Christian P.

Klocko, Andrew D.

Kobor, Michael S.

Kocher, Thomas D.

Koehler, Simone

Koenig, Daniel

Kohwi, Minoree

Koide, Yohei

Kole, Chittaranjan

Koltes, James E.

Konstantinides, Nikos

Koonin, Eugene V.

Kopp, Artyom

Kopp, Michael

Kornfeld, Kerry

Korzh, Vladimir

Koseki, Haruhiko

Kosiol, Carolin

Koskela, Jere

Kosterlitz, Olivia

Kothe, Gregory O.

Koushika, Sandhya P.

Kover, Paula X.

Kratochwil, Claudius

Krauchunas, Amber

Krebber, Heike

Kretzschmar, Doris

Kronforst, Marcus R.

Kropp, Peter

Krug, Joachim

Kruth, Perryn S.

Kryazhimskiy, Sergey

Kubota, Takashi

Kudla, Grzegorz

Kudoh, Tetsuhiro

Kuhner, Mary

Kumar, Anuj

Kumar, Justin P.

Kumar, Sandeep

Kumar, Sharad

Kumsta, Caroline

Kuniyoshi, Daichi

Kupiec, Martin

Kuroyanagi, Hidehito

Kurshan, Peri

Kuzmin, Elena

Kuzminov, Andrei

Kwitek, Anne E.

Kyriacou, C.P.

Kyriazis, Chris

La Spada, Albert

LaBella, Abigail L.

Labroo, Marlee

Lagator, Mato

Lai, Eric

Laloë, D.

Lalonde, Christine

Lambert, Amaury

Lambie, Eric J.

Lamitina, Todd

Lammerding, Jan

Lang, Gregory I.

Lang, Michael

Lange, Leslie

Lansford, Rusty

Larracuente, Amanda M.

Larschan, Erica N.

Lasko, Paul

Lasky-Su, Jessica A.

Laudet, Vincent

Lawrence-Dill, Carolyn J.

Lawson, Heather A.

Lazarro, Brian

Lazo, Gerard R.

Lazzaro, Brian P.

Le Pabic, Pierre

Le Rouzic, Arnaud

Leamy, Larry J.

Lee, Heng-Chi

Lee, Jiae

Lee, Junho

Lee, Kichoon

Lee, Lindsay

Lee, Sang H.

Lee, Seung-been

Lee, Siu S.

Lee, Teresa

Leeb, Tosso

Legan, Andrew W.

Legarra, Andres

Legouis, Renaud

Legrand, Mélanie

Lehmann, Ruth

Lehner, Ben

Lemaitre, Bruno

Lenormand, Thomas

León-Sampedro, Ricardo

Leppälä, Kalle

Leppeck, Katrin

Lerit, Dorothy A.

Lesaffre, Thomas

Leube, Rudolf

Levine, Mia

Levy, Avraham A.

Lewis, Cathryn

Lewis, Zachary A.

Li, Fei

Li, Heng

Li, Huajin

Li, Huang

Li, Jingyi J.

Li, Miaoxin

Li, Wei Vivian

Li, Wenhao

Li, Xianghua

Li, Xuwen

Li, Yuekai

Li, Yun

Li, Ze

Li, Zenglu

Li, Zihao J.

Li, Zitong

Liang, Mei

Liao, Yi

Libri, Domenico

Libuda, Diana E.

Lichten, Michael

Lillie, Mette

Lilly, M.

Lim, Bomyi

Lima, Dayane C.

Lin, Zhenguo

Lindert, Steffen

Lindorff-Larsen, Kresten

Lipka, Alexander E.

Lis, John T.

Liti, Gianni

Little, Tom J.

Liu, Boxiang

Liu, Gaowen

Liu, Haoxuan

Liu, Kelly J.

Liu, Weifang

Liu, Xianan

Liu, Yie

Ljubojević, Mirjana

Lloret Fernández, Carla

Lobner-Olesen, Anders

Lock, Antonia

Lockwood, Brent

Loeb, Lawrence A.

Loewith, Robbie

Lohmar, Jessica M.

Lohmueller, Kirk E.

Lomate, Purushottam

Long, Anthony D.

Long, Manyuan

Lord, Kathryn

Lorrain, Cecile

Losick, Vicki P.

Loudet, Olivier

Louis, Edward J.

Lourenco, Daniela

Love, Michael

Lovering, Ruth C.

Lovett, Susan T.

Lowe-Power, Tiffany

Lowry, David B.

Lu, Ake t.

Lu, Hang

Lu, Zeyun

Lue, Neal

Luke, Brian

Luo, Kaixuan

Luo, Liqun

Luo, Yichen

Lusis, Aldons J.

Lysak, Martin A.

M'Angale, Peter

Ma, Dengke K.

Ma, Hong

Ma, Li-Jun

Ma, Yiming

Macabenta, Frank

MacAlpine, David

MacAlpine, Jessie

MacArthur, Michael

Macdonald, Stuart J.

Macdonald-dunlop, Erin

Macia, Javier

Mack, Katya

Mackay, Trudy F.

Mackintosh, Alexander

Mackintosh, Carl J.

MacPherson, Ailene

Madura, Kiran

Maduro, Morris F.

Magadi, Srivathsa S.

Maggert, Keith A.

Magwene, Paul M.

Mailand, Niels

Main, Dorrie

Maistrenko, Oleksandr

Maitra, Nairita

Majewski, Jacek

Makova, Kateryna

Malinsky, Milan

Maloof, Julin N.

Maltecca, Christian

Mancero-Castillo, Daniel A.

Mango, Susan

Mangone, Marco

Maniatis, Nikolas

Maniego, Jillian

Mank, Judith

Manrubia, Susanna

Marcogliese, Paul

Marcon, Caroline

Marioni, Ricardo

Marla, Sandeep R.

Marois, Eric

Marques, André

Marques, Joao

Marques da Cunha, Fernanda

Marques-Bonet, Tomas

Marrec, Loïc

Marschall, Tobias

Marsh, Joseph

Marshall-Colon, Amy

Marsit, Souhir

Marston, Adele L.

Marti, Emiliano

Martienssen, Rob

Martin, Arnaud

Martin, Chantel

Martin, Guillaume

Martin, Simon H.

Martin, Stanton

Martí­nez-Alesón Garcia, Paloma

Martiniano, Rui

Mary-Huard, Tristan

Mascher, Martin

Masel, Joanna

Maselko, Maciej

Masnovo, Chiara

Mason, Adam

Mata, Juan

Mathieson, Iain

Matos-Perdomo, Emiliano

Matschiner, Michael

Matsen IV, Frederick A.

Matunis, Erika

Mazo-Vargas, Anyi

Mbu'u, Cyrille M.

Mbuli-Robertson, Duncan

McCall, Kimberly

McCandlish, David

McCarthy, Fiona M.

McCay, Dan

McCutcheon, John P.

McDonald, Bruce A.

McDonald, Jeanne M.

McDonald, Megan

McFarland, Christopher

McGaugh, Suzanne

McGregor, Alistair

McGregor, Cecilia

McGrew, Michael

McGuigan, Katrina

McIntyre, Lauren M.

McJunkin, Katherine

McKay, Dan J.

McKee, Bruce D.

McKim, Kim S.

McKown, Athena

McLachlan, Ian G.

McManus, Joel

McMillan, W. Owen

McMullan, Mark

Meccariello, Angela

Medley, Jeffrey C.

Medwig-Kinney, Taylor N.

Mei, Xue

Meier, Joana I.

Meiklejohn, Colin D.

Meisel, Richard P.

Meisler, Miriam H.

Meissner, Alexander

Menda, Naama

Mendoza, Hector

Mendoza, Manuel

Menon, Mitra

Menon, Vaibhav D.

Mercier, Raphae

Merrill, Richard

Merritt, Thomas J.

Messer, Philipp W.

Messina, Giovanni

Metwally, Seifelden M.

Metzger, Brian P.

Meyer, Damon

Meyer, Joel

Meyer, Rhonda C.

Meyers, Lauren A.

Mezard, Christine

Miao, Jiacheng

Michaelson, Jake

Michaux, Grégoire

Mighton, Chloe

Milani, Lili

Millar, Neil

Miller, Danny E.

Miller, Matthew P.

Miller, Rachel K.

Min, Jiseon

Mishra, Shreya

Miska, Eric A.

Misztal, Ignacy

Mitchell, Aaron P.

Mitchell, Jennyfer M.

Mitina, Aleksandra

Mizumoto, Kota

Moineau, Sylvain

Moise, Alexander

Momen, Mehdi

Mondal, Jayanta

Monk, Kelly

Montesinos-López, Osval A.

Montgomery, Tai

Moore, Adrian W.

Moore, Claire

Moorjani, Priya

Moran, John V.

Morata, Gines

Moreira, Pedro

Morgan, Andrew P.

Morris, Andrew

Morrison, Maike L.

Morrow, Edward

Morschhäuser, Joachim

Mortimer, Nathan T.

Mosca, Timothy J.

Moseley, James

Mostafavi, Hakhamanesh

Mostoufi, Sabrina L.

Mott, Richard

Moyes, Catherine

Mueller, Lukas A.

Mugal, Carina F.

Mukherjee, Bhramar

Mukherjee, Shreyasi

Mullet, John

Mullis, Martin N.

Mullon, Charles

Munch, Kasper

Mungan, Muhittin

Munoz-Torres, Monica C.

Murakami, Yota

Muralidhar, Pavitra

Murphy, Coleen T.

Murthy, Mala

Musharoff, Shaila

Muszynski, Michael G.

Mutwil, Marek

Myers, Simon

Nagy, Laszlo

Naj, Adam

Nakhleh, Luay

Nance, Jeremy

Navarro, Arcadi

Navarro Mendoza, Maribel

Navarro-Costa, Paulo

Navon, Dina

Neher, Richard A.

Neiman, Aaron M.

Nelson, James C.

Nelson, Rex T.

Ness, Rob W.

Newcomb, Susan

Newton, Irene G.

Nguyen Ba, Alex N.

Nijhout, H.F.

Nijveen, Harm

Niwa, Ryusuke

Noble, Robert

Nolan, Tony

Nolan, Trevor

Nollen, Ellen

Noma, Ken-ichi

Noor, M. A.

Nordman, Jared

Norris, Megan L.

Novembre, John

Nunez, Joaquin C.

Nyangwara, Vincent

O'Connor-Giles, Kate M.

O'Grady, Patrick M.

O'Haren, Thomas

O'Keeffe, Catherine

Odom, Duncan

Oeffinger, Marlene

Ogbunugafor, Brandon

Olson, Bradley J.

Onn, Itay

Onogi, Akio

Opulente, Dana A.

Orenic, Teresa

Orive, Maria E.

Orr-Weaver, Terry L.

Ortega-Del Vecchyo, Diego

Ortlund, Eric A.

Osmond, Matthew M.

Oteo García, Gonzalo

Otto, Sarah P.

Ou, Shujun

Oughtred, Rose

Ozias-Akins, Peggy

Paaby, Annalise B.

Padeken, Padeken

Page-McCaw, Andrea

Paix, Alexandre

Pal, Arka

Pallares, Luisa

Palmer, Daniela

Palmer, Glen

Palmer Droguett, ​Daniela

Palsson, Bernhard

Pan, Yufeng

Pandey, Ashutosh

Panepinto, John

Paniagua, Nancy

Panning, Barbara

Papalopulu, Nancy

Papoutsoglou, Evangelia

Parée, Tom

Parichy, David M.

Park, Younshim

Parkhurst, Susan M.

Parkin, Isobel A.

Parkinson, John E.

Parrish, Jay

Parsons, Kevin

Pasero, Phillipe

Pashay Ahi, Ehsan

Pastor-Pareja, Jose C.

Patel, Nipam H.

Patel, Roshni A.

Paten, Benedict

Patterson, Nick J.

Pattillo Smith, Samuel

Pavlidis, Pavlos

Pawlowski, Wojciech P.

Payseur, Bret A.

Pazokitoroudi, Ali

Pearse, Devon E.

Pedro, Beltrao

Peede, David

Peichel, Catherine L.

Peischl, Stephan

Peixoto, Marco A.

Pellegrini, Matteo

Pellman, David

Peña-Miller, Rafael

Pennings, Pleuni S.

Pereira-Gómez, Marianoel

Perkin, Lindsey C.

Perry, Blair W.

Peter, Benjamin M.

Petes, Thomas D.

Petrella, Lisa N.

Peuget, Sylvain

Pfeifer, Susanne P.

Phadnis, Nitin

Phifer-Rixey, Megan

Philippe, Marullo

Phillips, Carolyn M.

Phillips, Patrick C.

Phillips, Sarah R.

Philpott, Anna

Piano, Fabio

Pick, Leslie

Piepho, Hans-Peter

Pierce, Jonathan T.

Pigliucci, Massimo

Pimentel, Harold

Pinto, Brendan J.

Pirinen, Matti

Pocrnic, Ivan

Pöggeler, Stefanie

Pollard, Katie

Pomiankowski, Andrew

Pommier, Cyril

Pomp, Daniel

Pomraning, Kyle R.

Pong-Wong, Ricardo

Pook, Torsten

Pool, John E.

Poole, Richard J.

Pope, Nathaniel

Popovic, Mina

Portman, Douglas

Posnien, Nico

Posth, Cosimo

Postlethwait, John H.

Potter, Christopher J.

Pouyet, Fanny

Powder, Kara E.

Powell, Joseph

Preger-Ben Noon, Ella

Presgraves, Daven C.

Prinz, Will

Program, Peer R.

Proko, Rinalda

Prokop, Andreas

Przeworski, Molly

Puri, Dharmendra

Qian, Wanqiang

Qin, Zhaohui

Quah, Fu Xiang

Quesneville, Hadi

Quickstad, Gabrielle

Quinlan, Aaron R.

Raab, Jesse R.

Radanović, Aleksandra

Raff, Jordan W.

Rafter, Pierce

Raghu, Padinjat

Raghuraman, M. K.

Ragsdale, Aaron P.

Rahbari, Raheleh

Rahit, K. M. Tahsin Hassan

Rai, Madhulika

Ralph, Peter L.

Ram, Yoav

Ramachandran, Sohini

Ramakers, Jip J.

Ramstein, Guillaume

Rangan, Prashanth

Rankin, Susannah

Rapti, Georgia

Rastas, Pasi

Rastegar, Sepand

Ratcliff, William

Ratnaparkhi, Girish S.

Ray, Rishav

Rebeiz, Mark

Rechtsteiner, Andreas

Recuerda, Maria

Rehkopf, David

Reiner, David J.

Reinke, Valerie

Reinthal, Peter N.

Reis, Tânia

Remsburg, Carolyn

Renz, Matthias

Rep, Martijn

Rera, Michael

Resende Jr., Marcio F.

Resnick, Jim

Rhen, Turk

Rhind, Nicholas

Ricci, Emiliano

Richmond, Janet E.

Rideout, Elizabeth

Ridges, Jackson T.

Rieder, Leila E.

Riffell, Jeffrey

Rihel, Jason

Rijiang, Zhou

Rincent, Renaud

Ringwald, Martin

Riquelme, Meritxell

Rissi, Daniel V.

Ritchie, Michael G.

Rivas-González, Iker

Robbins, Kelly R.

Robertson, R. M.

Robinson, Peter N.

Robinson-Rechavi, Marc

Robinson-Thiewes, Sarah

Rocha, Eduardo P.

Rochman, Nash

Rodriguez, Moira

Rodriguez Morales, Roberto E.

Rodríguez-Beltrán, Jerónimo

Roeder, G. S.

Rog, Ofer

Rogers, Alan R.

Rogers, Gregory

Rokas, Antonis

Roman, Gregg

Romanowski, Joseph S.

Roncoroni, Miguel

Rongo, Christopher

Röper, Katja

Rosbash, Michael

Rosenberg, Noah A.

Rosenberg, Susan M.

Rosenzweig, Benjamin

Rosling, Anna

Ross-Ibarra, Jeffrey

Roth, Frederick P.

Rothenberg, Katheryn

Rougvie, Ann E.

Rousselle, Marjolaine

Rousset, Francois

Roy, Sushmita

Rubin, Alan F.

Ruden, Douglas M.

Rudman, Seth M.

Runcie, Daniel E.

Rushlow, Christine

Russell, Shelbi

Ruvinsky, Ilya

Ruvkun, Gary

Ruzicka, Filip

Rydhmer, Lotta

Ryoo, Hyung Don

Sabatti, Chiara

Sachdeva, Himani

Sackton, Timothy

Sadli, Asli

Salas-Fernandez, Maria G.

Salehi, Sohrab

Salomon, Matthew

Saltz, Julia

Samocha, Kaitlin

Sampaio, Ana

Samuelson, David J.

Samuk, Kieran

San Millan, Alvaro

Sánchez, Juan A.

Sanderson, Lacey-Anne

Sanetra, Matthias

Sankaranarayanan, Sundar Ram

Santiago, Enrique

Santos, Emılia

Santos-Lopez, Alfonso

Sanyal, Kaustuv

Sardell, Jason M.

Sarsani, Vishal

Saski, Christopher

Satija, Rahul

Saunders, Scott H.

Sawa, Hitoshi

Saxena, Rahul

Scally, Aylwyn

Schacherer, Joseph

Schaeffer, Stephen W.

Schafer, William R.

Schedl, Paul

Schedl, Tim

Scheffler, Jodi

Schell, Bianca

Schierup, Mikkel H.

Schiffels, Stephan

Schimenti, John C.

Schirmeier, Stefanie

Schlenke, Todd A.

Schlogelhofer, Peter

Schlötterer, Christian

Schmid, Clemens

Schmidt, Heiko A.

Schmidt, Paul

Schmutz, Jeremy J.

Schnable, James C.

Schneemann, Hilde

Schoen, Hanna

Schofield, Paul N.

Scholz, Uwe

Schraiber, Joshua G.

Schrauf, Matias F.

Schroeder, Frank C.

Schuster-Böckler, Benjamin

Schwer, Beate

Scott, Jacob G.

Scott, Maxwell J.

Secomandi, Simona

Segev, Nava

Segu, Aishwarya

Segurado, MÃ³nica

Sehgal, Amita

Seidel, Hannah S.

Sekelsky, Jeff

Sella, Guy

Selmecki, Anna

Seluanov, Andrei

Sendrowski, Janek

Serio, Tricia

Seroude, Laurent

Serrano, Rita J.

Servedio, Maria

Sethi, Anurag

Setter, Derek

Seydoux, Geraldine

Shaack, Sarah

Shah, Premal

Shaikh, Razeen

Shannon, Laura M.

Sharma, Santosh

Sharma, Vartika

Sharp, Andrew

Sharp, Nathaniel P.

Shastry, Vivaswat

Shaulsky, Gad

Shaver, Amanda O.

Shaw, Ruth G.

Shaye, Daniel D.

Sheen, Jen

Sheets, Michael

Shen, Yufeng

Sherlock, Gavin

Sherwood, David

Shidlovskii, Yulii V.

Shimagaki, Kai S.

Shimizu, Kentaro K.

Shingleton, Alexander

Shirasawa, Kenta

Shoenhard, Hannah

Shore, David

Shorter, James

Shrestha, Padmina

Shulman, Joshua M.

Si, Kausik

Si, Yichen

Sibbesen, Jonas

Siddiq, Mohammad

Siddique, Kadambot H.

Sieburth, Derek

Siegal, Mark L.

Sierra, Pio

Simmons, Lyle

Simons, Yuval B.

Singh, Nadia D.

Singh, Pooja

Sinha, Himanshu

Sinha, Saurabh

Sinka, Rita

Sinsheimer, Janet S.

Skinner, Benjamin M.

Skovgaard, Ole

Skrutl, Lena

Slatkin, Montgomery

Slotte, Tanja

Small, Scott

Smith, Craig

Smith, Daniel J.

Smith, Jeffrey S.

Smith, Stephen

Smith-Bolton, Rachel K.

Smolikove, Sarit

Smukowski Heil, Caiti

Smulders, Marinus J.

Snowdon, Rod J.

Snyder-Mackler, Noah

Snyman, Marelize

Sogin, Maggie

Solberg Woods, Leah C.

Solé, Ricard

Solomon, Mark

Sommer, Ralf J.

Son, Jae Hak

Sonam, Surabhi

Song, Qijian

Song, Yun S.

Sørensen, Peter

Soto, Martha

Sousa Ferreira, Mafalda

Speed, Douglas

Speidel, Leo

Spence, Jeffrey P.

Spencer, Hamish G.

Spivakov, Mikhail

Sprando, Robert

Sreekumar, Lakshmi

St. Pierre, Celine L.

Staals, Raymond

Stadler, Tanja

Staller, Max V.

Stanfield, Gillian

Starr, Tyler

Staton, Margaret

Stearns, Frank

Steensma, Marije J.

Stefanakis, Nikolaos

Stein, Amelie

Stein, Nils

Steinrücken, Matthias

Stephan, Wolfgang

Stephens, Greg

Stephens, Matthew

Stern, David L.

Sternberg, Paul W.

Stetsenko, Roman

Stevens, Lewis

Stevison, Laurie S.

Stinchcombe, John

Stitzer, Michelle

Stote, Karen

Stottmann, Rolf

Stranden, Ismo

Strome, Erin D.

Stroustrup, Nicholas

Stukenbrock, Eva H.

Su, Chang

Su, Hang

Su, Tin Tin

Su, Ying

Sudmant, Peter H.

Sugihara, Yu

Sullivan, William

Sun, Huaigu

Sun, Jianjun

Sun, Lin

Sun, Quan

Sun, Wei

Sundaram, Meera V.

Sundararajan, Sanjana

Sutter, Nathan B.

Swaminathan, Kankshita

Sweigart, Andrea L.

Swinderen, Bruno

Swofford, David

Szpiech, Zachary A.

Taagen, Ella

Taberlay, Phillippa

Tagami, Takahiro Tagami

Takahashi, Kazuo H.

Takahashi, Yuma

Takeuchi, Nobuto

Tammineni, Eshwar Reddy

Tan, Hua L.

Tang, Chenwei

Tang, Ho Lam

Tang, Ho Man Holly

Tang, Wen

Tanny, Jason C.

Tao, Dayun

Tarnopol, Rebecca

Tarrant, Ann

Tarvin, Rebecca D.

Tate, Ann

Taubert, Stefan

Tautz, Diethard

Tavares, Álvaro A.

Taylor, Julian

Taylor, Martin

Taylor, Tiffany

Teichmann, Sarah

Temple, Seth

Templeton, Matthew D.

Tennessen, Jason M.

Terao, Chikashi

Terhorst, Jonathan

Thakar, Tanay

Thakur, Jitendra

Thapa, Suchitra

Thimmappa, Bhagya C.

Thomas, Ariane E.

Thomas, Gregg

Thomas, Laura

Thomas, Paul D.

Thomas, Peter

Thomson, Robert

Thornton, Joe

Thornton, Kevin R.

Threadgill, David W.

Thudi, Mahendar

Thummel, Carl S.

Tian, Chengzhe

Tissenbaum, Heidi

Tkačik, Gasper

Tokuriki, Nobuhiko

Tomkova, Marketa

Top, Eva M.

Toro, Miguel Angel

Torre, Matthew

Tournebize, Remi

Tourniaire, Julie

Tourrette, Elise

Tovar, Adelaide

Townsend, Jeffrey P.

Traven, Ana

Travisano, Michael

Trevisson, Eva

Trifunovic, Aleksandra

Troggio, Michela

Turelli, Michael

Tweedie, Susan

Tyrrell, Simon

Tzeng, Jung-Ying

Ubbens, Jordan

Udall, Joshua A.

Uecker, Hildegard

Ünal, Elçin

Unckless, Robert L.

Updike, Dustin L.

Uppuluri, Priya

Uricchio, Lawrence H.

Usher, Jane

Usman, Dawoud

Vaca, Luis

Valdar, William

Vale, Pedro

Valente, Bruno D.

Van Cleve, Jeremy

Van Kan, Jan

Van Werven, Folkert

Van Wijk, Klaas

Vandebergh, Marijne

Vanderpool, Dan

Varaldi, Julien

Vargas Jurado, Napoleón

Varona, Luis

Varshney, Gaurav

Varshney, Rajeev K.

Vaughen, John

Vaughn, Andrew H.

Vazquez, Ana I.

Véber, Amandine

Vega Alfaro, Andrey

Velayudhan, Shaji

Veller, Carl

Verbiest, Max

Verta, Jukka-Pekka

Vicoso, Beatriz

Villanea, Fernando A.

Visintin, Rosella

Visscher, Peter M.

Vitezica, Zulma G.

Vogt, Katrin

Volkan, Pelin C.

Vollger, Mitchell

Waagmester, Andra

Wacholder, Aaron

Wade, Claire M.

Wade, Paul A.

Wagner, Wolfgang

Wahl, Lindi M.

Wakeley, John

Walhout, Marian

Walker, Amy K.

Walker, Sarah

Walsh, Bruce

Walter, Greg

Wang, Jade

Wang, Liu

Wang, Shuang

Wang, Xiangfeng

Wang, Xutong

Wang, Yu-Feng

Wang, Yue

Wang, Zhiwei

Wang, Zigui

Wang, Ziyan

Waples, Robin

Wappner, Pablo

Ward, Jordan D.

Ware, Doreen

Ware, Stephanie

Washburn, Jacob D.

Wassarman, David A.

Wasserman, Wyeth W.

Wassmann, Katja

Waterhouse, Robert M.

Watts, Jennifer L.

Weber, Stephanie C.

Webster, Sarah E.

Wegrzyn, Jill L.

Wei, Hong-Jiang

Wei, Qing

Wei, Wei

Wei, Xinzhu

Weile, Jochen

Weinreich, Daniel

Weisman, Lois

Weissman, Daniel B.

Welch, John J.

Wellenreuther, Maren

Wellinger, Raymund J.

Welte, Michael

Wendland, Juergen

Wessells, Robert J.

Wessinger, Carrie

West, Jeffrey

West, Steven

Wharton, Kristi A.

Wheatley, Rachel

White, Benjamin

White-Cooper, Helen

Whitehead, Andrew

Whiteley, Andrew R.

Whiteman, Noah K.

Whiteway, Malcolm

Whiteway, Malcolm S.

Whitlock, Michael C.

Widder, Stefanie

Widjaja, Anissa

Wientjes, Yvonne C.

Wigby, Stuart

Wilkinson-Herbots, Hilde M.

Williams, Jacob

Williams, Robert W.

Williams, Scott M.

Williams, Suzanne

Willighagen, Egon

Wilson, Melissa A.

Wimmer, Valentin

Wingen, Luzie U.

Wingert, Rebecca

Wittenburg, Dörte

Wittkopp, Patricia J.

Wittmann, Meike J.

Wohl, Shirlee

Wohlever, Matthew

Wohns, Anthony W.

Wojcik, Genevieve

Wolde, Pieter Rein ten

Wolf, Jason B.

Wolfe, Kenneth H.

Wolfe, Marnin D.

Wölfl, Benjamin

Wolfner, Mariana F.

Wong, Yan

Woolford, John L.

Woollard, Alison

Wrasman, Kristie

Wray, Naomi R.

Wright, Alison

Wright, April

Wright, Stephen

Wu, Chong

Wu, Lang

Wu, Rongling

Wyrwoll, Margot

Xavier, Alencar

Xiang, Tao

Xiao, Jiashun

Xiao, Jingfa

Xie, Jeffrey

Xie, Ting

Xin, Zhanguo

Xu, Hong

Xu, Junxiang

Xu, Sen

Xu, Shizhong

Xu, Wei

Xu, Yuyan

Xu, Zhou

Yamashita, Yukiko M.

Yang, Can

Yang, Chin Jian

Yang, Hua

Yang, Jie

Yang, Jinliang

Yang, Yi

Yang, Zhikai

Yanowitz, Judith

Yao, Yuchen

Yemini, Eviatar

Yordanova, Martina

Yu, Fengwei

Yu, Jianming

Yuan, Quan

Yuan, Xiaojing

Yuan, Zhongshang

Yuh Chwen Lee, Grace

Yukilevich, Roman

Yuksel, Mete K.

Zaabza, Hafedh Ben

Zaidi, Arslan A.

Zang, Fuzhong

Zeggini, Eleftheria

Zeng, Jian

Zeng, Zhao-Bang

Zhang, Chao

Zhang, Guiquan

Zhang, Jianzhi

Zhang, Qianqian

Zhang, Qingrun

Zhang, Tianzhen

Zhang, Xinmi

Zhang, Yi

Zhang, Yun

Zhang, Zerui

Zhao, Tianjing

Zhao, Zhangwu

Zhao, Zhongying

Zheng, Chaozhi

Zheng, Xiaojing

Zhou, Jumin

Zhou, Lingbo

Zhou, Muqing

Zhou, Wenjun

Zhu, Yan

Zhuang, Xuan

Zielinski, Daniel

Ziolkowski, Piotr

Zitnik, Marinka

Zoller, Joseph A.

Zuryn, Steve

Zwanzig, Martin

